# Altered Expressions of Transfer RNA-Derived Small RNAs and microRNAs in the Vitreous Humor of Proliferative Diabetic Retinopathy

**DOI:** 10.3389/fendo.2022.913370

**Published:** 2022-07-12

**Authors:** Yan Yang, Wenyun Yue, Nan Wang, Zicong Wang, Bingyan Li, Jun Zeng, Shigeo Yoshida, Chun Ding, Yedi Zhou

**Affiliations:** ^1^ Department of Ophthalmology, The Second Xiangya Hospital of Central South University, Changsha, China; ^2^ Hunan Clinical Research Center of Ophthalmic Disease, Changsha, China; ^3^ Department of Ophthalmology, Kurume University School of Medicine, Kurume, Japan

**Keywords:** transfer RNA-derived small RNA, microRNA, proliferative diabetic retinopathy, retinal neovascularization, vitreous humor

## Abstract

**Purpose:**

We sought to reveal the expression profiles of transfer RNA-derived small RNAs (tsRNAs) and microRNAs (miRNAs) in the vitreous humor of patients with proliferative diabetic retinopathy (PDR).

**Methods:**

Vitreous humor samples were obtained from PDR patients and a control group for this study. Sequencing of small RNAs was conducted to assess the expression profiles of tsRNAs and miRNAs in both groups, which was followed by validation using reverse transcription-quantitative real-time polymerase chain reaction (RT-qPCR). Bioinformatics analyses were conducted to predict the target genes and their potential biological functions and signaling pathways.

**Results:**

A total of 37 tsRNAs and 70 miRNAs with significant differences were screened out from the vitreous humor samples of PDR patients compared to controls. Following validation by RT-qPCR, the target genes of the validated tsRNAs and miRNAs were predicted, and Gene Ontology analysis indicated that the target genes of the tsRNAs were most enriched in the cellular macromolecule metabolic process, cytoplasm, and ion-binding, while those of the miRNAs were most abundant in the regulation of major metabolic process, cytoplasm, and protein-binding. In addition, Kyoto Encyclopedia of Genes and Genomes pathway analysis showed that the target genes of said tsRNAs and miRNAs were most enriched in the adenosine monophosphate-activated protein kinase signaling pathway and Th17 cell differentiation, respectively.

**Conclusions:**

The present study identified altered tsRNAs and miRNAs in vitreous humor samples of PDR patients, which may play important roles in the pathogenesis of PDR and could be considered potential therapeutic targets in the treatment of PDR.

## Introduction

Diabetic retinopathy (DR) is a common complication of diabetes mellitus (DM), which has become a major cause of global blindness. With the aging of the population and the growing number of diabetic patients, DR has become a serious public health problem worldwide (1). As a more severe stage of DR, proliferative DR (PDR) is defined by retinal neovascularization, which may lead to serious loss of vision. Anti–vascular endothelial growth factor (VEGF) drugs have been widely applied clinically to inhibit pathological retinal neovascularization in PDR patients (2). However, these anti-VEGF drugs necessitate long-term repeated intravitreal injections, which greatly increases the medical economic burden. In addition, anti-VEGF drugs have a poor response in a considerable number of patients, as they are not capable of inhibiting all pathological neovascular tufts and may cause serious local and systemic complications in some cases (3). Ishikawa et al. identified gene-expression profile changes in the fibrovascular membranes of PDR patients and indicated that extracellular matrix–related molecules were involved in the development of said fibrovascular membranes (4). However, until now, the pathogenesis of PDR has remained unclear, and there is an urgent need to further explore the molecular mechanisms of retinal neovascularization in PDR and identify novel therapeutic targets with good safety and effectiveness so as to provide new methods for the diagnosis and treatment of PDR.

Recently, non-coding RNAs (ncRNAs) have emerged as research hotspots in medical and molecular biological studies. Although ncRNAs do not have the capability to translate genes to proteins, their regulatory roles in various physiological and pathological processes cannot be ignored (5). Small ncRNAs are important ncRNAs measuring < 200 nucleotides in length that include many types of RNAs, such as microRNAs (miRNAs), transfer RNA–derived small RNAs (tsRNAs), small nuclear RNAs, small nucleolar RNAs, and Piwi-interacting RNAs (6).

It has been reported that numerous miRNAs are involved in the development of DR (7). For instance, miR-15b attenuates angiogenesis by targeting VEGF (8), while miR-18a-5p inhibits retinal neovascularization *via* the downregulation of *FGF1* and *HIF1A* (9). On the other hand, another study indicated that miR-409-5p enhances retinal angiogenesis in DR (10).

Unlike miRNAs, tsRNAs are a novel type of small ncRNA that have been much less frequently explored. tsRNAs are derived from transfer RNAs and play regulatory roles in many biological processes and diseases (11, 12). According to the varying cleavage positions of precursor or mature tRNA transcripts, tsRNAs can be classified into 2 main subtypes, which are tRNA-derived fragments and tRNA-derived stress-induced RNAs (11). It has been revealed that tsRNAs contribute to different mechanisms in cancer, neurological disorders, and viral infections and can also serve as biomarkers for disease diagnosis and prognosis (13). We previously identified alterations in tsRNA expression profiles in mouse models of laser-induced choroidal neovascularization (14) and oxygen-induced retinopathy (OIR) (15). Although the OIR mouse model is widely used in the investigation of hypoxia-induced retinal neovascular diseases (16), the process of OIR modeling is very different from the clinical situation of PDR pathogenesis. Zhang et al. compared expression profiles of circular RNAs (circRNAs) in vitreous humor samples of patients with PDR and non-DM patients (17). Therefore, it is meaningful to reveal the expression profiles and possible functions of tsRNAs in clinical samples (such as vitreous humor samples) collected from patients with PDR.

In this study, we collected vitreous humor samples from PDR patients and controls, who were idiopathic macular hole (IMH) patients without DM. To identify the altered tsRNAs and miRNAs in samples from PDR patients, small RNA sequencing was conducted followed by validation with reverse transcription-quantitative real-time polymerase chain reaction (RT-qPCR). In addition, bioinformatics analyses were performed to predict the target genes and their potential biological functions and signaling pathways.

## Materials and Methods

### Collection of Clinical Samples

Vitreous humor samples were collected from PDR patients and non-DM patients with IMH during plana vitrectomy surgeries. We diagnosed PDR by recognizing retinal neovascular tufts, vitreous hemorrhage, and fibrovascular membranes. Samples were collected from patients with severe vision loss because of PDR-induced vitreous hemorrhage, aged 50-70 years old, and patients were excluded when they had (1) PDR combined with neovascular glaucoma and rhegmatogenous retinal detachment; (2) a history of other vitreoretinal diseases, such as retinal vascular occlusion and age-related macular degeneration; (3) a history of intraocular surgery, laser treatment, or intravitreal injection medicine (including anti-VEGF and dexamethasone); (4) a history of systemic disease, including infectious, inflammatory, autoimmune, or immunosuppressive diseases and uncontrolled hypertension; (5) a history of myocardial infarction or cerebrovascular accidents, and coagulation abnormality or current use of an anticoagulative medication. The exact conditions and surgical indications of the included PDR patients were listed in **Supplementary Table 1**. Non-DM patients with IMH were considered to be the control group. A total of 1 mL of vitreous humor was collected from each individual, then stored in a refrigerator at -80°C after a quick freeze in liquid nitrogen.

This study adhered to the tenets of the Declaration of Helsinki, and the protocol of this study was approved by the ethics committee of the Second Xiangya Hospital of Central South University (number: 2021LY037). Informed consent was obtained from all participants before their recruitment into the study.

### RNA Isolation From the Vitreous Humor

RNA was isolated from vitreous humor samples using a Trizol RNA extraction kit (Invitrogen, Carlsbad, CA, USA). The integrity and quantity of each RNA sample were assessed by agarose gel electrophoresis and NanoDrop ND-1000 spectrophotometry (Thermo Fisher Scientific, Waltham, MA, USA).

### Library Preparation, Small RNA Sequencing, and Data Analysis

The protocols of library preparation, small RNA sequencing, and data analysis were adopted as previously described (14). In brief, first, total RNA was sequentially ligated to 3’ and 5’ small RNA adapters for each sample. Second, complementary DNA (cDNA) synthesis and amplification were conducted using Illumina’s proprietary RT primers and amplification primers. Third, PCR-amplified fragments (~134–160 bp) were extracted from the polyacrylamide gel electrophoresis gel and purified. Finally, the completed libraries were quantified by the Agilent 2100 bioanalyzer (Agilent Technologies, Santa Clara, CA, USA). The libraries were denatured and diluted to a loading volume of 1.3 mL and loading concentration of 1.8 pM. Diluted libraries were loaded onto a reagent cartridge and forwarded for a sequencing run on the Illumina NextSeq 500 system using the NextSeq 500/550 V2 kit (Illumina, San Diego, CA, USA). Raw sequencing data, which were generated from the Illumina NextSeq 500 and passed the Illumina chastity filter, were used for the following analysis, and we also calculated the expression profiles of tsRNAs and miRNAs. The raw data of the small RNA sequencing were deposited in Gene Expression Omnibus database (Accession No. GSE199852).

### Validation by RT-qPCR

The small RNA sequencing results were validated by RT-qPCR. To assess the expression of tsRNAs, RNA pre-treatment was performed by using the rtStar™ tRF and tiRNA pre-treatment kit (Arraystar, Rockville, MD, USA); then, the samples were transcribed into cDNA utilizing rtStar™ First-Strand cDNA Synthesis Kit (3’ and 5’ adaptor) (Arraystar). RT-qPCR was performed using 2 × PCR Master Mix (Arraystar) on the QuantStudio™ 5 real-time PCR system (Applied Biosystems, Foster City, CA, USA).

To evaluate miRNA expression levels, RNA was transcribed into cDNA using M-MuLV reverse transcriptase (Enzymatics, Beverly, MA, USA) as directed by the manufacturer with the Gene Amp PCR system 9700 (Applied Biosystems). RT-qPCR was performed using the ViiA 7 real-time PCR system (Applied Biosystems) with 2 × PCR Master Mix (Arraystar).

The reaction conditions of the 2 experiments were as follows: incubation at 95°C for 10 min, incubation at 95°C for 10 s, and incubation at 60°C for 60 s, with 40 cycles. Expression levels were calculated with a 2−ΔΔCt method (18) and normalized with U6. Primer sequences are shown in **Tables 1**, **2**.

**Table 1:** The sequences of primers used for RT-qPCR validation of tsRNAs.

tsRNA	Primer sequence	Tm (°C)	Product length (bp)
U6	F:5’GCTTCGGCAGCACATATACTAAAAT3’ R:5’CGCTTCACGAATTTGCGTGTCAT3’	60	89
tRF-57:75-Pro-AGG-1-M7	F:5’AGTCCGACGATCAATCCCG3’ R:5’CTCTTCCGATCTTGGGGGC3’	60	43
tRF-1:24-Val-AAC-1-M7	F:5’CGATCGTTTCCGTAGTGTAGTG3’ R:5’GTGTGCTCTTCCGATCTTGATA3’	60	46
tRF-+1:T16-Thr-TGT-2	F:5’CGACGATCCCTGTTGGCTTA3’ R:5’GACGTGTGCTCTTCCGATCTAA3’	60	44
tRF-56:72-chrM.Val-TAC	F:5’AGTTCTACAGTCCGACGATCCTT3’ R:5’TTCCGATCTTGGTCAGAGCG3’	60	46
tRF-57:76-Tyr-GTA-1-M2	F:5’CTACAGTCCGACGATCGAATCC3’ R:5’GCTCTTCCGATCTTGGTCCTT3’	60	49
tRF-55:76-Arg-ACG-1-M2	F:5’GATCTCGACTCCTGGCTGGC3’ R:5’TGTGCTCTTCCGATCTTGGC3’	60	42

**Table 2:** The sequences of primers used for RT-qPCR validation of miRNAs.

miRNA	Primer sequence	Tm (°C)	Product length (bp)
U6	F:5’GCTTCGGCAGCACATATACTAAAAT3’ R:5’CGCTTCACGAATTTGCGTGTCAT3’	60	89
hsa-miR-889-3p	GSP:5’GGGGGTTAATATCGGACAAC3’ R:5’GTGCGTGTCGTGGAGTCG3’	60	64
hsa-miR-939-5p	GSP:5’GTGGGGAGCTGAGGCTCT3’ R:5’GTGCGTGTCGTGGAGTCG3’	60	63
hsa-miR-1469	GSP:5’AATCTCGGCGCGGGG3’ R:5’GTGCGTGTCGTGGAGTCG3’	60	63
hsa-miR-4755-3p	GSP:5’GCAGCCAGGCTCTGAAGG3’ R:5’GTGCGTGTCGTGGAGTCG3’	60	62
hsa-miR-411-5p	GSP:5’GGACAGCAGACCGCACAG3’ R:5’GTGCGTGTCGTGGAGTCG3’	60	62
hsa-miR-369-3p	GSP:5’GGGGAATAATACATGGTTG R:5’CAGTGCGTGTCGTGGA3’	60	65
hsa-miR-181d-5p	GSP:5’GGGGCATTCATTGTTGTCG3’ R:5’GTGCGTGTCGTGGAGTCG3’	60	63
hsa-miR-125a-5p	GSP:5’GCTCCCTGAGACCCTTTA3’ R:5’CAGTGCGTGTCGTGGAGT3’	60	66

### Target Gene Prediction, Gene Ontology (GO), and Kyoto Encyclopedia of Genes and Genomes (KEGG) Pathway Analyses

The target prediction databases of miRanda and TargetScan were used to predict the target genes of validated miRNAs, and the common algorithms were used to predict the target genes of validated tsRNAs. The GO (http://www.geneontology.org/) and KEGG (http://www.genome.jp/kegg/) databases were used to predict the potential biological functions and involved pathways of the target genes.

### Statistical Analyses

Data from small RNA sequencing were evaluated using counts per million reads mapped (CPM). The CPM values of the PDR group and the control group were calculated using a negative binomial generalized linear model, and *p* values were evaluated using a negative binomial distribution. A fold change ≥ 1.5 and *p <* 0.05 were used to screen for altered tsRNAs and miRNAs. RT-qPCR results were expressed as mean ± standard error of the mean values, and Student’s *t*-test was used to evaluate the statistical difference. In all cases, *p* < 0.05 was considered to be statistically different.

## Results

### Clinical Characteristics of the Enrolled Subjects

Eight patents were included in this study, including 4 PDR patients and 4 non-DM patients with IMH as controls, respectively. The demographic features of the recruited patients are shown in **Table 3**. There was no significant difference in age, gender, body mass index, or intraocular pressure between the groups. However, the fasting blood glucose level in the PDR group was significantly higher than that of the control group. All patients in the PDR group had type 2 diabetes with an average disease duration of 12.50 ± 3.70 years, and the glycated hemoglobin value of the PDR group was 8.20% ± 1.75%.

**Table 3:** Clinical characteristics of included subjects of the study.

Characteristics	PDR Group (n=4)	Control Group (n=4)	P-value
Age (y)	58.00 ± 4.83	59.75 ± 6.85	0.691
Gender (male/female)	2/2	2/2	–
Diabetic duration (y)	12.50 ± 3.70	n.a.	–
BMI (kg/m^2^)	21.77 ± 4.48	23.03 ± 4.25	0.698
Fasting blood glucose (mmol/L)	8.02 ± 1.45	4.94 ± 0.23	0.006**
HbA1c%	8.20 ± 1.75	n.a.	–
IOP (mmHg)	17.00 ± 1.41	17.00 ± 1.41	1.000

BMI, Body Mass Index; HbA1c, glycated hemoglobin; IOP, Intraocular Pressure. **p < 0.01, na; no applicable.

### Catalog of tsRNA Expressions in Vitreous Humor Samples

**Figure 1:**
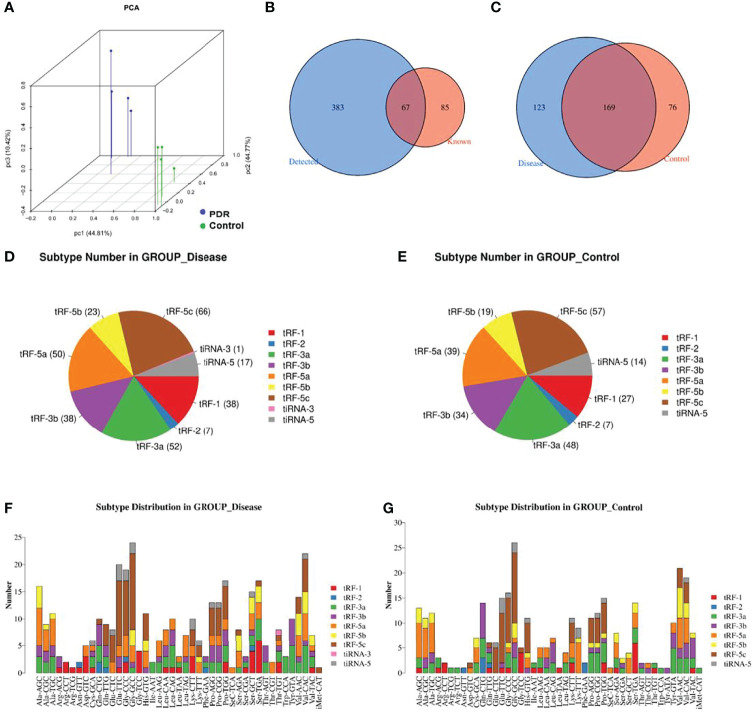
Catalog of tsRNA expressions in vitreous humor samples. **(A)** Primary component analysis was performed by principal component analysis, which was conducted using tsRNAs. **(B)** The Venn diagram shows the number of detected tsRNAs and the known tsRNAs. **(C)** The Venn diagram shows the number of commonly expressed and specifically expressed tsRNAs in the PDR (disease) and control groups. **(D, E)** Pie charts display the numbers of different tsRNA subtypes in the PDR **(D)** and control **(E)** groups. **(F, G)** Different subtype distributions of tsRNAs in the PDR **(F)** and control **(G)** groups.

A principal component analysis plot was used to overview the small RNA sequencing data and revealed an obvious distinction between the groups (**Figure 1A**). The Venn diagram in **Figure 1B** demonstrates that, in total, 450 kinds of tsRNAs were detected in this study, of which 67 tsRNAs are known from the tRFdb database. As shown in **Figure 1C**, 169 tsRNAs were commonly expressed in both PDR and control samples, 123 tsRNAs were only expressed in PDR samples, and 76 were only expressed in control samples (CPM ≥ 20).

The pie chart shows the distribution of each expressed tsRNA subtype (**Figures 1D, E**). A total of 38 tRF-1, 7 tRF-2, 52 tRF-3a, 38 tRF-3b, 50 tRF-5a, 23 tRF-5b, 66 tRF-5c, 1 tiRNA-3, and 17 tiRNA-5 RNA(s) were identified in the PDR group (**Figure 1D**). On the other hand, 27 tRF-1, 7 tRF-2, 48 tRF-3a, 34 tRF-3b, 39 tRF-5a, 19 tRF-5b, 57 tRF-5c, and 14 tiRNA-5 RNAs were identified in the control group, while no tiRNA-3 RNA was recognized (**Figure 1E**). The numbers of subtype tsRNAs against tRNA isodecoders of the PDR group and control group are also shown (**Figures 1F, G**).

### Altered Expression Profiles of tsRNAs and miRNAs in Vitreous Humor Samples of PDR Patients

To reveal the expression alterations of tsRNAs and miRNAs in the vitreous humor samples from PDR patients, a threshold of a fold change ≥ 1.5 and *p* < 0.05 were applied to identify the significantly different expressions.

**Figure 2:**
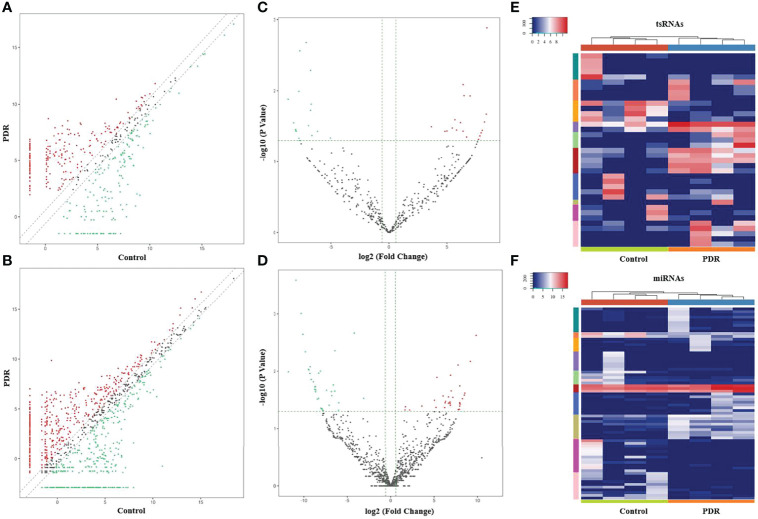
Altered expression profiles of tsRNAs and miRNAs in vitreous humor samples from PDR patients compared to controls. **(A, B)** The scatterplots of altered tsRNAs **(A)** and miRNAs **(B)**. The red and green dots represent the up- and down-regulated tsRNAs/miRNAs, respectively (fold change ≥ 1.5). **(C, D)** The volcano plots of significantly altered tsRNAs **(C)** and miRNAs **(D)**. The red and green dots represent the significantly up- and down-regulated tsRNAs/miRNAs, respectively (fold change ≥ 1.5, *p* < 0.05). **(E, F)** A heatmap and hierarchical clustering analysis of significantly altered tsRNAs **(E)** and miRNAs **(F)** in vitreous humor samples of the PDR and control groups.

The variation in tsRNA expression levels between the PDR and control groups is shown as a scatterplot (**Figure 2A**) and a volcano plot (**Figure 2C**). The results indicated that 20 tsRNAs were up-regulated and 17 were down-regulated in the PDR group compared to the control group (**Figure 2E**). The top 10 up- and down-regulated tsRNAs are listed in **Table 4**. In particular, tRF-1:22-chrM.Ser-GCT and tRF-+1:T24-Leu-AAG-1 were the most significantly up- and down-regulated tsRNAs in the PDR group, respectively.

**Table 4:** Top 10 up- and down-regulated tsRNAs in vitreous humor samples of PDR patients.

tsRNA	Type	Length	Regulation	log2FC	P-value
tRF-1:22-chrM.Ser-GCT	tRF-5b	22	up	8.443880	0.001304
tRF-+1:T25-Leu-CAG-1-6	tRF-1	25	up	8.389052	0.021170
tRF-1:31-Ser-AGA-1-M6	tRF-5c	31	up	8.227896	0.027329
tRF-61:77-Ile-AAT-2-M3	tRF-3a	17	up	8.032453	0.035820
tRF-1:14-chrM.Ala-TGC	tRF-5a	14	up	7.939212	0.038880
tRF-56:77-Thr-AGT-3	tRF-3b	22	up	7.838917	0.042854
tiRNA-1:20-chrM.Ser-GCT	tiRNA-5	20	up	7.829087	0.043066
tRF-1:16-Leu-TAA-1	tRF-5a	16	up	7.715198	0.047983
tRF-57:76-Tyr-GTA-1-M2	tRF-3b	20	up	6.988874	0.011771
tRF-68:86-Leu-CAG-1	tRF-3b	19	up	6.693507	0.044548
tRF-+1:T24-Leu-AAG-1	tRF-1	24	down	-8.688740	0.013120
tRF-1:14-Tyr-GTA-1-M7	tRF-5a	14	down	-8.215016	0.028057
tRF-56:75-Thr-CGT-2-M2	tRF-3b	20	down	-8.058716	0.034978
tRF-56:75-Gln-CTG-2	tRF-3b	20	down	-8.035050	0.035697
tRF-55:75-Gln-TTG-1-M2	tRF-3b	21	down	-7.998365	0.036983
tRF-1:32-Gly-TCC-3	tRF-5c	32	down	-7.757781	0.010194
tRF-66:86-Leu-CAG-1	tRF-3b	21	down	-7.756151	0.046285
tRF-58:76-Tyr-ATA-1	tRF-3b	19	down	-7.679754	0.049904
tRF-56:72-chrM.Val-TAC	tRF-3a	17	down	-7.679407	0.002737
tRF-1:28-Glu-TTC-2	tRF-5c	28	down	-7.138055	0.002093

In addition, the variation in miRNA expression levels between the PDR and control groups is shown as a scatterplot (**Figure 2B**) and a volcano plot (**Figure 2D**). As shown in the heatmap, 34 and 36 miRNAs were significantly increased and decreased in the PDR samples, respectively (**Figure 2F**). The top 10 up- and down-regulated miRNAs are listed in **Table 5**. According to the fold changes, hsa-miR-6734-5p and hsa-miR-1297 were the most significantly up- and down-regulated miRNAs in the PDR group, respectively.

**Table 5:** Top 10 up- and down-regulated miRNAs in vitreous humor samples of PDR patients.

miRNA	Length	Regulation	log2FC	P-value
hsa-miR-6734-5p	23	up	9.899317	0.002366
hsa-miR-4755-3p	22	up	9.221942	0.006720
hsa-miR-518f-5p	22	up	8.592911	0.024083
hsa-miR-137-3p	23	up	8.523897	0.026054
hsa-miR-369-5p	22	up	8.305219	0.029994
hsa-miR-6877-5p	22	up	8.050634	0.037353
hsa-miR-1287-5p	22	up	8.022825	0.036348
hsa-miR-6794-5p	20	up	8.010205	0.017993
hsa-miR-642b-3p	22	up	7.999533	0.035298
hsa-miR-1297	17	up	7.998397	0.040450
hsa-miR-4316	17	down	-11.741562	0.010238
hsa-miR-1183	27	down	-10.871765	0.000252
hsa-miR-11401	20	down	-10.267196	0.000981
hsa-miR-7156-5p	23	down	-10.073930	0.002273
hsa-miR-520g-5p	23	down	-9.785546	0.004598
hsa-miR-5693	22	down	-9.448693	0.009282
hsa-miR-501-5p	22	down	-9.374017	0.009450
hsa-miR-99a-3p	22	down	-9.260094	0.010906
hsa-miR-1323	22	down	-9.232822	0.008348
hsa-miR-2278	22	down	-9.115166	0.013852

### Validation of the Altered tsRNAs and miRNAs by RT-qPCR

**Figure 3:**
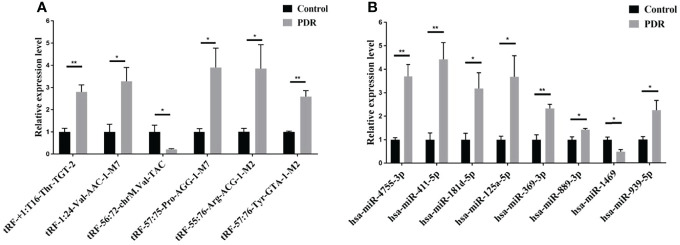
RT-qPCR validation of the significantly altered tsRNAs and miRNAs. **(A)** Relative expression levels of the tsRNAs by RT-qPCR. **(B)** Relative expression levels of the miRNAs by RT-qPCR. *, *p* < 0.05; **, *p* < 0.01.

To validate the reliability of the small RNA sequencing, RT-qPCR was performed to assess the expressions of selected tsRNAs and miRNAs. As shown in **Figure 3A**, tRF-57:75-Pro-AGG-1-M7, tRF-1:24-Val-AAC-1-M7, tRF-+1:T16-Thr-TGT-2, tRF-57:76-Tyr-GTA-1-M2, and tRF-55:76-Arg-ACG-1-M2 were significantly increased in the PDR samples compared to the control samples, while tRF-56:72-chrM.Val-TAC was significantly decreased. In addition, the expression levels of hsa-miR-889-3p, hsa-miR-939-5p, hsa-miR-4755-3p, hsa-miR-411-5p, hsa-miR-369-3p, hsa-miR-181d-5p, and hsa-miR-125a-5p were significantly enhanced, while that of hsa-miR-1469 was dramatically attenuated in the PDR samples (**Figure 3B**). The results of RT-qPCR indicated that the trend of these changes were similar to the results of the small RNA sequencing.

### Target Gene Prediction

To illustrate the prediction of ncRNA-messenger RNA (mRNA) interaction, target gene networks of the validated tsRNAs and miRNAs were constructed according to the databases (miRanda and TargetScan). In total, there were 2,950 target genes associated with the 6 validated tsRNAs by 3,195 edges (**Supplementary Figure 1**), while 700 target genes were associated with 7 of the 8 altered miRNAs by 721 edges (**Supplementary Figure 2**).

### GO and KEGG Analyses of the Target Genes

**Figure 4:**
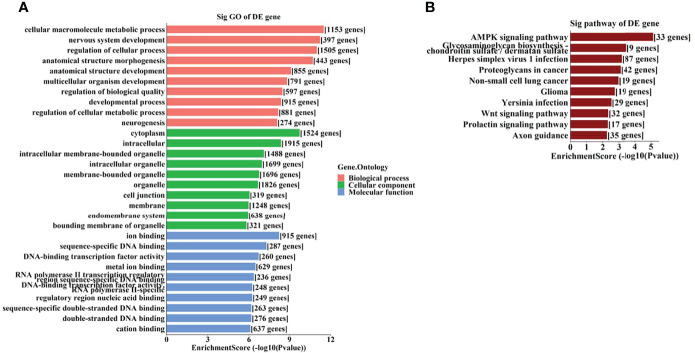
The GO and KEGG analyses by target genes of tsRNAs. The GO analysis **(A)** and KEGG pathway analysis **(B)** of targets based on 6 validated tsRNAs.

**Figure 5:**
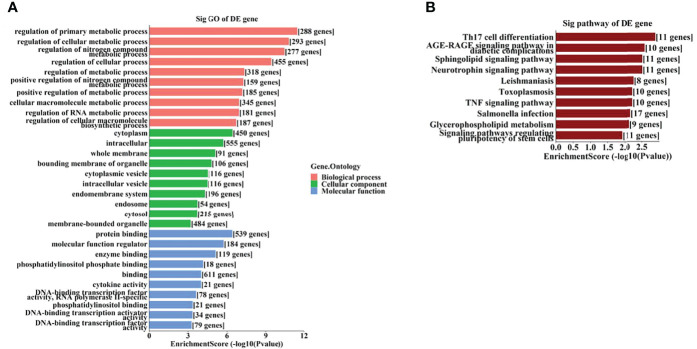
The GO and KEGG analyses by target genes of miRNAs. The GO analysis **(A)** and KEGG pathway analysis **(B)** of targets based on 7 validated tsRNAs.

To further identify the potential biological functions and involved signaling pathways of the predicted target genes mentioned above, GO and KEGG analyses were performed. The GO analysis indicated that the target genes of these 6 tsRNAs were most enriched in the cellular macromolecule metabolic process, cytoplasm, and ion-binding (**Figure 4A**), while the target genes of the 7 miRNAs were most enriched in the regulation of primary metabolic process, cytoplasm, and protein-binding (**Figure 5A**).

On the other hand, the KEGG pathway analysis demonstrated that the target genes of those tsRNAs were most enriched in the adenosine monophosphate–activated protein kinase (AMPK) signaling pathway (**Figure 4B**), while those of tsRNAs were most enriched in Th17 cell differentiation (**Figure 5B**).

## Discussion

Evidence has accumulated that ncRNAs, including miRNAs, long ncRNAs (lncRNAs), and circRNAs, have an essential role in the occurrence and development of DR (19). Several miRNAs, such as hsa-miR-3184-3p, hsa-miR-24-3p, and hsa-miR-197-3p, are overexpressed in the vitreous humor of PDR patients, and intravitreal injection of anti-VEGF drugs inhibits the increase of these miRNAs (20). Despite the direct regulatory actions of target genes on mRNA expression levels, miRNAs also serve as targets of the competitive endogenous RNA (ceRNA) network, which is constructed using lncRNAs or circRNAs (21). For example, knockdown of the lncRNA TUG1 inhibited the migration and tube-formation capabilities of retinal microvascular endothelial cells under high glucose-stimulation by targeting miR-145; in other words, lncRNA TUG1 serves as a ceRNA by sponging miR-145 (22).

Compared to miRNAs, tsRNAs are novel and remain more unknown. It has been reported that the tsRNA tRF-Gly-GCC is involved in the pathogenesis of atherosclerosis *via* functional regulation in human umbilical vein endothelial cells and vascular smooth muscle cells (23). Until now, few studies have investigated the role of tsRNAs in ocular disorders.

In this study, the expression profiles of tsRNAs and miRNAs were assessed in vitreous humor samples of PDR and control patients by small RNA sequencing, followed by RT-qPCR. In addition, bioinformatics analysis revealed the predicted biological functions and involved pathways of the target genes.

As GO analysis indicated (**Figures 4A**, **5A**), metabolic processes were involved in the target genes of both tsRNAs and miRNAs. Our previous study reported different levels of metabolites (especially amino acids and their derivatives) in retinal tissues of an OIR mouse model, and the impact on the metabolite changes may be a long-term one (24). It has been demonstrated that the metabolic profiles of plasma and vitreous humor are significantly altered in PDR patients (25–27). Thus, it is interesting to further study the correlations of altered tsRNAs/miRNAs and the changes in metabolites in PDR.

AMP-activated protein kinase (AMPK) pathway activation is a well-known mechanism which plays dual roles in angiogenesis (28). AMPK pathway also regulates metabolism and protects cells from pathogenic alterations in DR (29). It has been reported that the AMPK signaling pathway is involved in the etiopathogenesis of many ocular diseases, including DR (30). Song et al. demonstrated that stimulation of AMPK signaling pathway attenuate diabetes-induced photoreceptor degeneration through regulation of autophagy and mitochondrial function (31). AMPK phosphorylation is required in the regulation of HMGB1 induced by Epac1 in the diabetic retinal vasculature (32). In this study, we identified the AMPK signaling pathway as a leading enriched pathway associated with the altered tsRNAs (**Figure 4B**), which also suggested that these tsRNAs might have regulatory functions in the pathogenesis of DR.

It has been revealed that the Th17 immune response as well as the related cytokines (such as IL-17 and IL-23) play important regulatory roles in retinal neovascular diseases (33, 34). Th17 immune response may also be involved in promoting functional and morphological changes in retinas of spontaneously developing diabetes (35). Th17 cell differentiation is a leading enriched pathway of the target genes of the altered miRNAs in this study, it is also interesting to explore the potential roles of these miRNAs associated with Th17 immune response.

This is an innovational study to report the expression profiles of tsRNAs from human clinical samples of PDR, which indicated a huge alteration of tsRNAs in the vitreous humor of PDR patients. Moreover, this study also predicted the target genes of the significantly changed tsRNAs, as well as the potential biological functions and involved pathways, which shed light on future investigations into the roles and mechanisms of those specific tsRNAs in PDR pathogenesis.

Limitations exist in this study. First, IMH patients were included as the control group, and their vitreous humor might be different from the normal population. Second, it is necessary to increase the number of included subjects to validate the altered tsRNAs and miRNAs. Third, functional experiments are necessary to further explore the roles and mechanisms of the altered tsRNAs and miRNAs in PDR pathogenesis.

In conclusion, the present study indicated the altered expression profiles of tsRNAs and miRNAs, and the significantly altered tsRNAs and miRNAs, as well as the predicted signaling pathways, might be involved in the development and pathogenesis of PDR. Therefore, these tsRNAs/miRNAs have a potential for clinical application and could become novel therapeutic targets in the treatment of PDR.

## Data Availability Statement

The datasets presented in this study can be found in online repositories. The names of the repository/repositories and accession number(s) can be found below: GEO, GSE199852.

## Ethics Statement

The studies involving human participants were reviewed and approved by the ethics committee of the Second Xiangya Hospital of Central South University. The patients/participants provided their written informed consent to participate in this study. Written informed consent was obtained from the individual(s) for the publication of any potentially identifiable images or data included in this article.

## Author Contributions

YZ and CD conceived and designed the study. YY, YZ, CD, and BL analyzed the data and wrote the manuscript. YY, WY, NW, ZW, and JZ obtained the samples and clinical records. JZ and SY reviewed and revised the manuscript. All authors contributed to the article and approved the final version of the manuscript.

## Funding

This work was supported by National Natural Science Foundation of China (No. 81800855), Natural Science Foundation of Hunan Province (No. 2021JJ40885), Scientific Research Project of Hunan Provincial Health Commission (No. 202207022574), Development Project of Hunan Development and Reform Commission (No. 2021-212), and New Technology Incubation Funds in Ophthalmology.

## Conflict of Interest

The authors declare that the research was conducted in the absence of any commercial or financial relationships that could be construed as a potential conflict of interest.

## Publisher’s Note

All claims expressed in this article are solely those of the authors and do not necessarily represent those of their affiliated organizations, or those of the publisher, the editors and the reviewers. Any product that may be evaluated in this article, or claim that may be made by its manufacturer, is not guaranteed or endorsed by the publisher.

## Supplementary Material

Supplementary Figure 1 The network of validated tsRNAs and the target genes. The pink diamond nodes represent tsRNAs, the green circular nodes represent target genes (mRNAs), and the T-shaped edges represent targets.

Supplementary Figure 2 The network of validated miRNAs and the target genes. The pink diamond nodes represent miRNAs, the green circular nodes represent target genes (mRNAs), the gray lines represent targets without experimental validation by miRTarBase7.0, and the blue lines represent targets with experimental validation by miRTarBase7.0.

## References

[1] Sabanayagam C Banu R Chee M L Lee R Wang Y X Tan G. Incidence and Progression of Diabetic Retinopathy: A Systematic Review. Lancet Diabetes Endocrinol (2019) 7(2):140–9. doi: 10.1016/S2213-8587(18)30128-1 30005958

[2] Osaadon P Fagan X J Lifshitz T Levy J. A Review of Anti-VEGF Agents for Proliferative Diabetic Retinopathy. Eye (Lond) (2014) 28(5):510–20. doi: 10.1038/eye.2014.13 PMC4017101 24525867

[3] Salam A Mathew R Sivaprasad S. Treatment of Proliferative Diabetic Retinopathy With Anti-VEGF Agents. Acta Ophthalmol (2011) 89(5):405–11. doi: 10.1111/j.1755-3768.2010.02079.x 21294854

[4] Ishikawa K Yoshida S Kobayashi Y Zhou Y Nakama T Nakao S. Microarray Analysis of Gene Expression in Fibrovascular Membranes Excised From Patients With Proliferative Diabetic Retinopathy. Invest Ophthalmol Vis Sci (2015) 56(2):932–46. doi: 10.1167/iovs.14-15589 25604687

[5] Patil VS Zhou R Rana TM. Gene Regulation by non-Coding RNAs. Crit Rev Biochem Mol Biol (2014) 49(1):16–32. doi: 10.3109/10409238.2013.844092 24164576 PMC4721600

[6] Watson CN Belli A Di Pietro V. Small Non-Coding RNAs: New Class of Biomarkers and Potential Therapeutic Targets in Neurodegenerative Disease. Front Genet (2019) 10:364. doi: 10.3389/fgene.2019.00364 31080456 PMC6497742

[7] Satari M Aghadavod E Mirhosseini N Asemi Z. The Effects of microRNAs in Activating Neovascularization Pathways in Diabetic Retinopathy. J Cell Biochem (2019) 120(6):9514–21. doi: 10.1002/jcb.28227 30556195

[8] Yang Y. MicroRNA-15b Targets VEGF and Inhibits Angiogenesis in Proliferative Diabetic Retinopathy. J Clin Endocrinol Metab (2020) 105(11):3404–15. doi: 10.1210/clinem/dgaa538 PMC7947967 32797181

[9] Guan J T Li X X Peng D W Zhang W M Qu J Lu F. MicroRNA-18a-5p Administration Suppresses Retinal Neovascularization by Targeting FGF1 and HIF1A. Front Pharmacol (2020) 11:276. doi: 10.3389/fphar.2020.00276 32210827 PMC7076186

[10] Wang Y Lin W Ju J. MicroRNA-409-5p Promotes Retinal Neovascularization in Diabetic Retinopathy. Cell Cycle (2020) 19(11):1314–25. doi: 10.1080/15384101.2020.1749484 PMC7469530 32292119

[11] Li S Xu Z Sheng J. tRNA-Derived Small RNA: A Novel Regulatory Small Non-Coding RNA. Genes (Basel) (2018) 9(5):246. doi: 10.3390/genes9050246 PMC5977186 29748504

[12] Shen Y Yu X Zhu L Li T Yan Z Guo J. Transfer RNA-Derived Fragments and tRNA Halves: Biogenesis, Biological Functions and Their Roles in Diseases. J Mol Med (Berl) (2018) 96(11):1167–76. doi: 10.1007/s00109-018-1693-y 30232504

[13] Jia Y Tan W Zhou Y. Transfer RNA-Derived Small RNAs: Potential Applications as Novel Biomarkers for Disease Diagnosis and Prognosis. Ann Transl Med (2020) 8(17):1092. doi: 10.21037/atm-20-2797 33145311 PMC7575943

[14] Zhang L Liu S Wang J H Zou J Zeng H Zhao H. Differential Expressions of microRNAs and Transfer RNA-Derived Small RNAs: Potential Targets of Choroidal Neovascularization. Curr Eye Res (2019) 44(11):1226–35. doi: 10.1080/02713683.2019.1625407 31136199

[15] Peng Y Zou J Wang J H Zeng H Tan W Yoshida S. Small RNA Sequencing Reveals Transfer RNA-Derived Small RNA Expression Profiles in Retinal Neovascularization. Int J Med Sci (2020) 17(12):1713–22. doi: 10.7150/ijms.46209 PMC7378657 32714074

[16] Connor KM Krah NM Dennison RJ Aderman CM Chen J Guerin KI. Quantification of Oxygen-Induced Retinopathy in the Mouse: A Model of Vessel Loss, Vessel Regrowth and Pathological Angiogenesis. Nat Protoc (2009) 4(11):1565–73. doi: 10.1038/nprot.2009.187 PMC3731997 19816419

[17] He M Wang W Yu H Wang D Cao D Zeng Y. Comparison of Expression Profiling of Circular RNAs in Vitreous Humour Between Diabetic Retinopathy and non-Diabetes Mellitus Patients. Acta Diabetol (2020) 57(4):479–89. doi: 10.1007/s00592-019-01448-w 31749049

[18] Livak KJ Schmittgen TD. Analysis of Relative Gene Expression Data Using Real-Time Quantitative PCR and the 2(T)(-Delta Delta C) Method. Methods (2001) 25(4):402–8. doi: 10.1006/meth.2001.1262 11846609

[19] Chang X Zhu G Cai Z Wang Y Lian R Tang X. miRNA, lncRNA and circRNA: Targeted Molecules Full of Therapeutic Prospects in the Development of Diabetic Retinopathy. Front Endocrinol (Lausanne) (2021) 12:771552. doi: 10.3389/fendo.2021.771552 34858342 PMC8631471

[20] Guo J Zhou P Liu Z Dai F Pan M An G. The Aflibercept-Induced MicroRNA Profile in the Vitreous of Proliferative Diabetic Retinopathy Patients Detected by Next-Generation Sequencing. Front Pharmacol (2021) 12:781276. doi: 10.3389/fphar.2021.781276 34938191 PMC8685391

[21] Virciglio C Abel Y Rederstorff M. Regulatory Non-Coding RNAs: An Overview. Methods Mol Biol (2021) 2300:3–9. doi: 10.1007/978-1-0716-1386-3_1 33792866

[22] Shi Q Tang J Wang M Xu L Shi L. Knockdown of Long Non-Coding RNA TUG1 Suppresses Migration and Tube Formation in High Glucose-Stimulated Human Retinal Microvascular Endothelial Cells by Sponging miRNA-145. Mol Biotechnol (2022) 64(2):171–7. doi: 10.1007/s12033-021-00398-5 34554391

[23] He X Yang Y Wang Q Wang J Li S Li C. Expression Profiles and Potential Roles of Transfer RNA-Derived Small RNAs in Atherosclerosis. J Cell Mol Med (2021) 25(14):7052–65. doi: 10.1111/jcmm.16719 PMC8278088 34137159

[24] Zhou Y Tan W Zou J Cao J Huang Q Jiang B. Metabolomics Analyses of Mouse Retinas in Oxygen-Induced Retinopathy. Invest Ophthalmol Vis Sci (2021) 62(10):9. doi: 10.1167/iovs.62.10.9 PMC8363770 34374743

[25] Wang H Li S Wang C Wang Y Fang J Liu K. Plasma and Vitreous Metabolomics Profiling of Proliferative Diabetic Retinopathy. Invest Ophthalmol Vis Sci (2022) 63(2):17. doi: 10.1167/iovs.63.2.17 PMC8842420 35133401

[26] Sun Y Zou H Li X Xu S Liu C. Plasma Metabolomics Reveals Metabolic Profiling For Diabetic Retinopathy and Disease Progression. Front Endocrinol (Lausanne) (2021) 12:757088. doi: 10.3389/fendo.2021.757088 34777253 PMC8589034

[27] Tomita Y Cagnone G Fu Z Cakir B Kotoda Y Asakage M. Vitreous Metabolomics Profiling of Proliferative Diabetic Retinopathy. Diabetologia (2021) 64(1):70–82. doi: 10.1007/s00125-020-05309-y 33099660 PMC7718434

[28] Li Y Sun R Zou J Ying Y Luo Z. Dual Roles of the AMP-Activated Protein Kinase Pathway in Angiogenesis. Cells (2019) 8(7):752. doi: 10.3390/cells8070752 PMC6678403 31331111

[29] Hsu SK Cheng KC Mgbeahuruike MO Lin YH Wu CY Wang HD. New Insight Into the Effects of Metformin on Diabetic Retinopathy, Aging and Cancer: Nonapoptotic Cell Death, Immunosuppression, and Effects Beyond the AMPK Pathway. Int J Mol Sci (2021) 22(17):9453. doi: 10.3390/ijms22179453 34502359 PMC8430477

[30] Shukal DK Malaviya PB Sharma T. Role of the AMPK Signalling Pathway in the Aetiopathogenesis of Ocular Diseases. Hum Exp Toxicol (2022) 41:9603271211063165. doi: 10.1177/09603271211063165 35196887

[31] Song S Bao S Zhang C Zhang J Lv J Li X. Stimulation of AMPK Prevents Diabetes-Induced Photoreceptor Cell Degeneration. Oxid Med Cell Longev (2021) 2021:5587340. doi: 10.1155/2021/5587340 34093959 PMC8140850

[32] Jiang Y Steinle JJ. Epac1 Requires AMPK Phosphorylation to Regulate HMGB1 in the Retinal Vasculature. Invest Ophthalmol Vis Sci (2020) 61(11):33. doi: 10.1167/iovs.61.11.33 PMC7500149 32940662

[33] Li Y Zhou Y. Interleukin-17: The Role for Pathological Angiogenesis in Ocular Neovascular Diseases. Tohoku J Exp Med (2019) 247(2):87–98. doi: 10.1620/tjem.247.87 30773517

[34] Sui A Chen X Yao Y Yao Y Shen X Zhu Y. The IL-23/IL-17 Axis Promotes the Formation of Retinal Neovascularization by Activating the NLRP3 Inflammasome in Macrophages in an Experimental Retinopathy Mouse Model. Immunology (2021) 164(4):803–16. doi: 10.1111/imm.13402 PMC8561108 34396536

[35] Taguchi M Someya H Inada M Nishio Y Takayama K Harimoto K. Retinal Changes in Mice Spontaneously Developing Diabetes by Th17-Cell Deviation. Exp Eye Res (2020) 198:108155. doi: 10.1016/j.exer.2020.108155 32717339

