# Supplementary figures and images for: Altered Expressions of Transfer RNA-Derived Small RNAs and microRNAs in the Vitreous Humor of Proliferative Diabetic Retinopathy
Source: Front Endocrinol (Lausanne). 2022 Jul 12;13:913370. doi: 10.3389/fendo.2022.913370 (PMC9315217; doi:10.3389/fendo.2022.913370)

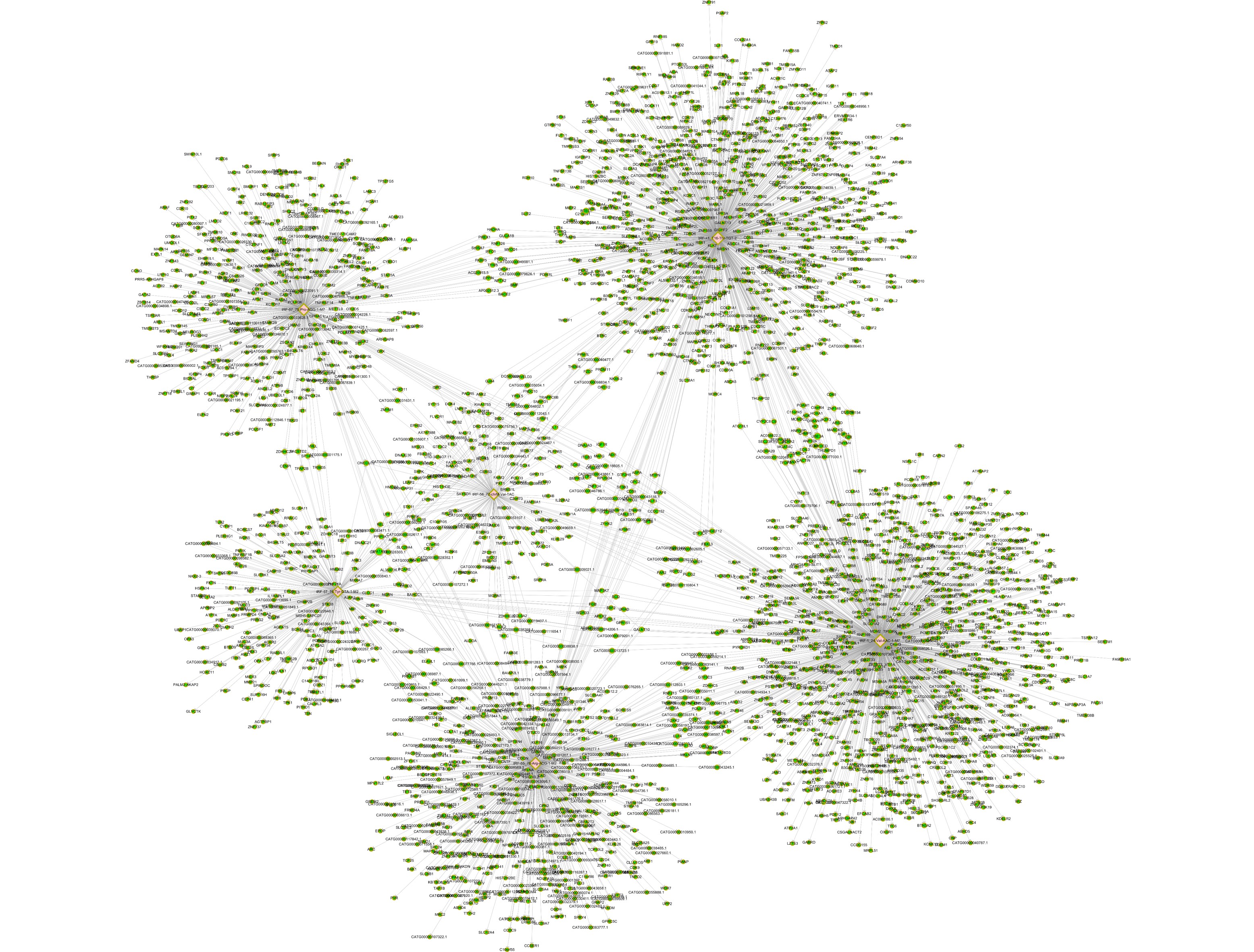

Supplement: Supplementary Figure 1 — The network of validated tsRNAs and the target genes. The pink diamond nodes represent tsRNAs, the green circular nodes represent target genes (mRNAs), and the T-shaped edges represent targets. [file Image_1.jpeg]

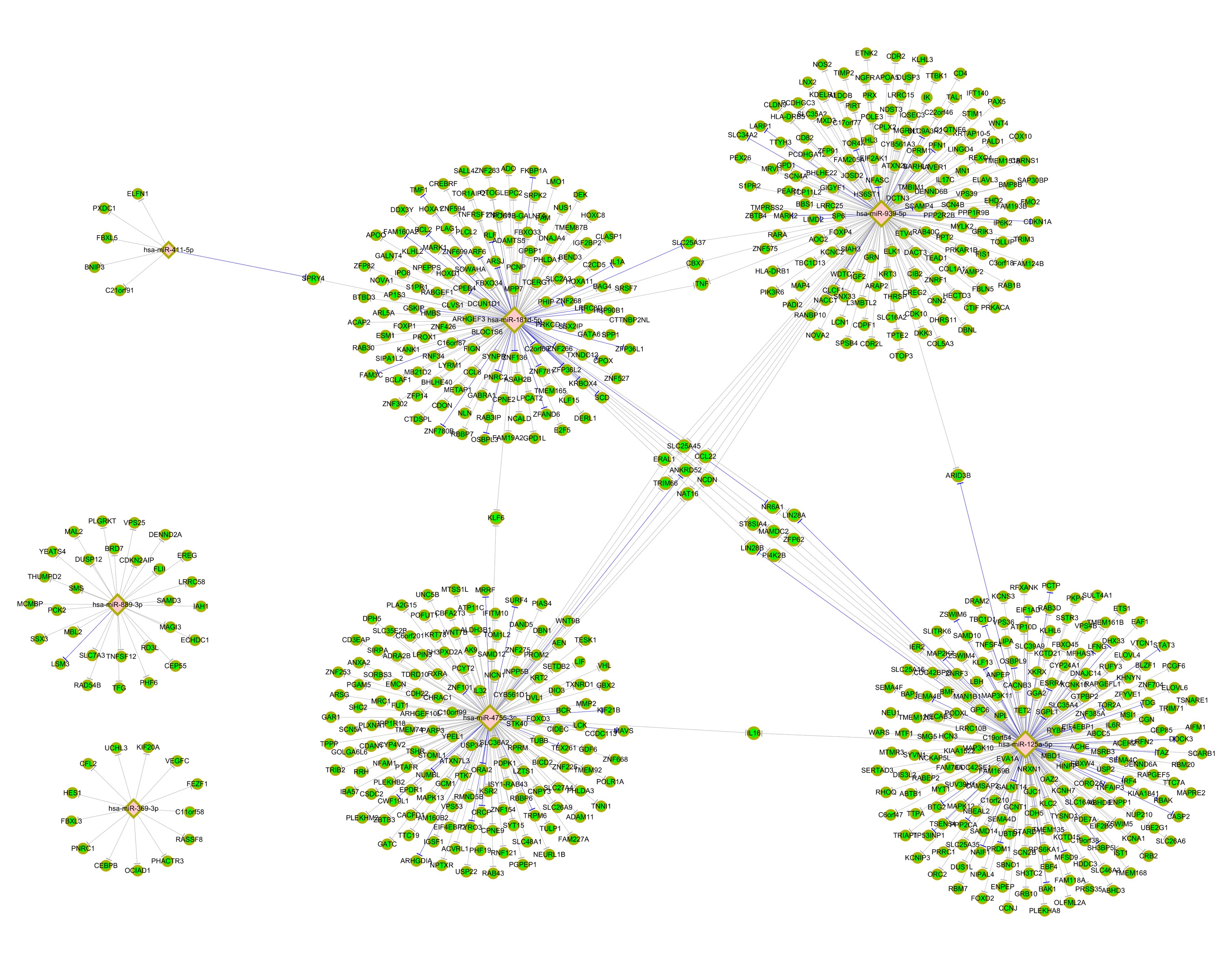

Supplement: Supplementary Figure 2 — The network of validated miRNAs and the target genes. The pink diamond nodes represent miRNAs, the green circular nodes represent target genes (mRNAs), the gray lines represent targets without experimental validation by miRTarBase7.0, and the blue lines represent targets with experimental validation by miRTarBase7.0. [file Image_2.jpeg]
